# Effects of dietary supplementation of *Poria cocos* polysaccharides on intestinal barrier, immune function, and growth of Hyla rabbits

**DOI:** 10.3389/fvets.2025.1643620

**Published:** 2025-11-07

**Authors:** Wen-Yi Wei, XIao-Peng Zhou, XIao-Lin Yang, Ying Zong, Kun Shi, Jian-Ming Li, Nai-Chao Diao, Rui Du, Fan-Li Zeng

**Affiliations:** 1 College of Chinese Medicinal Materials, Jilin Agricultural University, Changchun, China; 2 Jilin Province Sika Deer Efficient Breeding and Product Development Technology Engineering Research Center, Changchun, China; 3 The Ministry of Education Key Laboratory of Animal Production and the Product Quality and Safety, Changchun, China

**Keywords:** Hyla rabbit, growth performance, antioxidant, immunity function, gut microbiome, *Poria cocos* polysaccharide

## Abstract

**Introduction:**

Optimal rabbit health, which significantly influences growth and development, depends on three key factors: a robust immune system, proper intestinal function, and balanced gut microbiota. *Poria cocos* polysaccharide (PCP), the primary bioactive component of *Poria cocos*, exhibits multiple pharmacological properties with demonstrated benefits for animal health.

**Methods:**

320 Hyla rabbits were randomly allocated to four dietary groups: a control group receiving a basal diet and three experimental groups supplemented with 0.1, 0.2%, or 0.3% PCP. The growth performance of the rabbits was measured on day 21 and day 42. At the end of the experimental period, growth performance was evaluated, and samples of serum, thymus, liver, spleen, kidney, duodenum, cecum, and cecal content were collected. These samples were used to assess serum biochemical parameters, antioxidant capacity, organ indices, immune function, intestinal permeability, intestinal morphology, microbial composition, and short-chain fatty acid (SCFA) concentrations.

**Results:**

The results showed that the PCP supplementation significantly enhanced growth performance and immune organ indices in Hyla rabbits. Compared with the control group, PCP was able to significantly increase serum levels of total protein (*p* < 0.05), albumin (*p* < 0.05), glucose (*p* < 0.05), total antioxidant capacity (*p* < 0.05), catalase (*p* < 0.05), glutathione peroxidase (*p* < 0.01), Immunoglobulin A (*p* < 0.05), Immunoglobulin G (*p* < 0.001), Immunoglobulin M (*p* < 0.01), and Interleukin-10 (*p* < 0.01), and down-regulate serum levels of total cholesterol (*p* < 0.05), triglyceride (*p* < 0.05), malondialdehyde (*p* < 0.01), Interleukin-6 (*p* < 0.05), diamine oxidase, D-lactate, and endotoxin (*p* < 0.05). And PCP significantly increased villus length (*p* < 0.05) and villus-to-crypt ratio (*p* < 0.01), as well as duodenum-related intestinal gene expression (*p* < 0.05) in the duodenum and cecum, and decreased crypt depth in the duodenum and cecum (*p* < 0.01). In addition, PCP significantly increased the concentration of short-chain fatty acids and improved the structure of gut microbiota.

**Conclusion:**

In conclusion, these data suggest that PCP can be used as a potential tool to enhance growth performance by improving serum biochemistry, antioxidant capacity, immunity, gut barrier function, and gut flora composition in Hyla rabbits.

## 1 Introduction

Rabbit farming has several advantages, including low costs, high returns, a short breeding cycle, and flexible breeding times. Moreover, rabbit meat is rich in polyunsaturated fatty acids and protein, while being low in fat and cholesterol, offering high nutritional value (1). This makes it well-suited to meet people’s demand for a healthy diet, contributing to the rapid development of the meat rabbit industry both domestically and internationally. Farmers usually wean meat rabbits at 35 days after birth to improve the economic efficiency of their farms. Weaning is a critical period in a rabbit’s life and presents the most difficult challenges in the rearing of young rabbits (2). Weaning stress significantly compromises intestinal barrier integrity in rabbits, resulting in dysbiosis, immune dysregulation, and endocrine dysfunction (3). These pathophysiological alterations exacerbate oxidative stress, impair growth performance, and increase disease susceptibility, ultimately leading to elevated morbidity and mortality that adversely impact rabbit farming productivity (4, 5).

The intestinal tract serves as a vital digestive organ and the largest immune organ in young rabbits, playing a crucial role in digestion, absorption, metabolism, and immunity (6). Therefore, maintaining optimal intestinal barrier and function is of paramount importance for the growth, development, metabolic processes, and immune defense of young rabbits. Until now, alleviating weaning stress and controlling diarrhea and other intestinal diseases in rabbits has been mainly dependent on the use of antibiotics (7). However, concerns about antibiotic resistance, residual hazards (8), and the enforcement of ‘antibiotic-free’ policies across numerous countries have necessitated the exploit of green and natural alternatives to antibiotics that are safe and efficient to enhance the immune system and balance of intestinal microbiota in rabbits, thereby guaranteeing the healthy development of the livestock at the present stage.

In recent years, polysaccharides derived from traditional Chinese medicine (TCM) have gained widespread application as eco-friendly feed additives in livestock and poultry production, owing to their remarkable pharmacological properties in enhancing intestinal health, immune function, and growth performance (9). Poria cocos is a widely recognized functional edible and medicinal fungus, with polysaccharides serving as its primary bioactive component. Studies have revealed that *Poria cocos* polysaccharide (PCP) exhibits a range of bioactivities, including anti-inflammatory, antioxidant, antitumor, and immunomodulatory effects (10, 11). In animal production, Zhang et al. (12) discovered that PCP (phytochemical compound, presumed for context) positively impacts weaned piglets by enhancing their growth performance, boosting immunity, and modulating cecum microbiota composition, ultimately promoting piglet health. Zhang et al. (13) reported that incorporating PCP into the diet of LPS-challenged broilers alleviated intestinal inflammation and mucosal damage, thereby enhancing their performance. Duan et al. (14) demonstrated that PCP functions as a functional food by regulating the intestinal mucosa and barrier function in mice, which helps maintain intestinal homeostasis, consequently, enhances their health. Nevertheless, the application of PCP has been predominantly investigated in animals such as pigs and chickens. Consequently, its effects on antioxidant capacity, immune responses, and intestinal barrier integrity in Hyla weaned rabbits remain to be elucidated.

Thus, this study aims to investigate the effects of dietary supplementation with varying levels of PCP on growth performance, antioxidant capacity, immune function, intestinal barrier integrity, and gut microbiota composition in Hyla rabbits.

## 2 Materials and methods

### 2.1 *Poria cocos* polysaccharide preparation

*Poria cocos* polysaccharide (PCP) was obtained from the Shanghai Yuanye Bio-Technology Co., Ltd. (Beijing, China) as a grayish-white powder with a drying weight loss of less than 5% and a polysaccharide content of ≥50%.

### 2.2 Animal, experimental design, and dietary management

A total of 320 healthy 35-day-old weaned Hyla meat rabbits (average body weight: 710 g), comprising equal numbers of males and females, were obtained from the Shangzhi Experimental Rabbit Breeding Base in Harbin for this study. The rabbits were randomly allocated into 4 groups (8 replicates per group, 10 rabbits per replicate). Based on a comprehensive review of relevant literature and preliminary experimental data, the dietary supplementation levels of PCP were determined to be 0.1, 0.2, and 0.3%. Rabbits were divided into four groups: a control group (CON) fed a basal diet without PCP, and three treatment groups supplemented with 0.1% (PL), 0.2% (PM), or 0.3% (PH) PCP in the basal diet. The test rations were formulated with reference to the Nutritional Requirements of Meat Rabbits (NY/T4049-2021) as pelleted feed, and their composition and nutritional levels are shown in Table 1. The experimental period lasted for 49 days, including a 7-day pretrial period. The first 7 days served as the pretrial period, during which the rabbits adapted to the new housing, new group, and reduced weaning stress, and were only fed with the basic diet. The following 42 days constituted the main trial period. The test rabbits were housed in upper and lower double-layer cages and fed twice a day with free water during the trial. All rabbits had completed routine vaccination before weaning, and the rabbit hutches were regularly disinfected throughout the experiment. The composition and nutritional levels of the basic diet are shown in Table 1.

**Table 1:** Composition and nutrient levels of basal diets (air-dried basis, %).

Ingredient	Content, %	Nutrient levels^b^	Content, %
Corn	12.0	Metabolizable energy (ME, MJ/kg)	10.5
Alfalfa pellets	36.0	Crude protein (CP, %)	18.5
Wheat bran	17.0	Crude fiber (CF, %)	14.0
Wheat middlings	5.0	Neutral detergent fiber (NDF, %)	34.0
Soybean meal	13.5	Acid detergent fiber (ADF, %)	20.0
Corn germ meal	10.0	Calcium (Ca, %)	1.0
CaHPO₄	0.5	Total phosphorus (TP, %)	0.60
Limestone	1.0	Lysine (Lys, %)	0.80
NaCl	0.2	Methionine+Cystine (Met+Cys, %)	0.60
Premix^a^	4.0		
Total	100		

a The premix provided the following per kg of the basal diet: vitamin A 10000 IU; vitamin D_3_ 1,200 IU; vitamin E 50 mg; vitamin K_3_ 2 mg; vitamin B_1_ 2 mg; vitamin B_2_ 4 mg; vitamin B_6_ 2 mg; vitamin B_12_ 0.02 mg; icotinic acid 50 mg, Calcium pantothenate 20 mg; Folic acid 1 mg; Biotin 0.1 mg; Choline chloride 500 mg; Fe (as ferrous sulfate) 80 mg; Cu (as copper sulfate) 10 mg; Zn (as zinc sulfate) 60 mg; Mn (as manganese sulfate) 15 mg; I (as potassium iodide) 0.5 mg; Se (as sodium selenite) 0.3 mg.

b Digestible energy was calculated, the rest were measured values.

### 2.3 Growth performance

At the beginning and end of the experiment, the rabbits were weighed, with the recordings labeled as initial body weight (IBW) and final body weight (FBW). During the experiment, daily feed consumption was recorded, and average daily gain (ADG), average daily feed intake (ADFI), and feed-to-gain ratio (F/G) were calculated.

### 2.4 Sample collection

On the day the experiment ended, one rabbit was randomly selected from each repeat of each group for sterile ear marginal vein blood collection. The blood was then centrifuged at 3,000 r/min at 4 °C for 10 min to separate the serum, which was subsequently stored at −20 °C for future use. Euthanasia of rabbits was performed in accordance with the 2020 American Veterinary Medical Association (AVMA) Guidelines for the Euthanasia of Animals. The abdominal cavity was then immediately opened to excise the thymus, spleen, liver, kidneys, and round vesicles. After blotting the surface blood with absorbent paper, organs were weighed and subsequently stored at −80 °C. The relative weights of immune organs were calculated using the following formula: Immune organ index (g/kg) = organ weight (g) /body weight (kg). Tissue samples were collected and preserved in 4% paraformaldehyde fixative from the duodenum and cecum (approximately 3 cm). The contents of the duodenum and cecum were collected for microbiota analysis, the intestines were rinsed with sterile saline, and the mucosa of the washed intestinal sections was then gently scraped using a sterilised microscope slide for gene expression analysis. All samples were stored at −80 °C.

### 2.5 Serum biochemical analysis

Serum biochemical indicators including protein (TP), albumin (ALB), globulin (GLB), glucose (GLU), urea nitrogen (BUN), lactate dehydrogenase (LDH), alkaline phosphatase (ALP), total cholesterol (TC), triglyceride (TG) were tested by using the relevant kit from Nanjing Jiancheng Bioengineering Institute, China.

### 2.6 Determination of antioxidant indicators

Serum and liver antioxidant capacities, including total antioxidant capacity (T-AOC), catalase (CAT), glutathione peroxidase (GSH-Px), superoxide dismutase (SOD), and malondialdehyde (MDA) were measured using commercial kits from Nanjing Jiancheng Bioengineering Institute, China.

### 2.7 Determination of immune function

Serum levels of immunoglobulin A (IgA), immunoglobulin G (IgG), immunoglobulin M (IgM), interferon-γ (IFN-γ), interleukin-2 (IL-2), interleukin-6 (IL-6), and interleukin-10 (IL-10), as well as duodenum levels of secretory immunoglobulin A (SIgA), IgG, and IL-10, were measured using a commercial solid-phase sandwich ELISA kit (Shanghai Enzyme-linked Biotechnology Co., Ltd., China).

### 2.8 Histological observation of the duodenum and cecum

The tissue specimens were fixed in 4% paraformaldehyde solution, followed by dehydration and paraffin embedding. Serial sections of 5-μm thickness were prepared and stained with hematoxylin and eosin (H&E). For microscopic analysis, six well-oriented fields of view were randomly selected from each section. The intestinal mucosal morphology was examined, with measurements of villus height (VH) and crypt depth (CD) recorded. Villus height (VH) was measured as the vertical distance from the villus tip to the crypt-villus junction, whereas crypt depth (CD) was determined as the perpendicular distance from the crypt-villus junction to the base of the crypt. The villus-to-crypt ratio (V/C) was subsequently calculated.

### 2.9 Intestinal permeability analysis

The serum diamine oxidase (DAO), endotoxin, and D-lactate tested using commercial assay kits (Shanghai Optimal Biotechnology Co. Ltd., China).

### 2.10 Intestinal mucosal gene expression

Total RNA was extracted from duodenum samples using the Trans-Zol UP Plus RNA Extraction Kit (TransGen Biotech, Beijing, China) following the manufacturer’s protocol. RNA concentration and purity were determined before reverse transcription into cDNA, which was stored at −20 °C for subsequent analysis. Quantitative real-time PCR (qPCR) was performed using PerfectStart® Green qPCR Super Mix (TransGen Biotech) with gene-specific primers (designed and synthesized by Sangon Biotech, Shanghai, China; sequences listed in Table 2). The thermal cycling conditions consisted of initial denaturation at 94 °C for 30 s, followed by 40 cycles of denaturation at 94 °C for 5 s, annealing at 56 °C for 15 s, and extension at 72 °C for 10 s. GAPDH was used as the reference gene for normalization, and relative gene expression was calculated using the 2^−ΔΔCT^ method.

**Table 2:** Primer sequences for quantitative real-time PCR.

Gene	Primer sequence (5 → 3)	GeneBank
GADPH	F:GCACGGTCAAGGCTGAGAACG	NM_001082253.1
	R:GCACCAGCATCACCCCACTTG	
Occludin	F:TTGAGCAGCAGCAGTAACTTTGAG	XM_008262318.4
	R:GGTCGTGTAGTCCGTCTCGTAG	
Claudin-1	F:CCCTGCCTCAATGGAAGATTTACTC	NM_001089316.1
	R:GCTCTGCGACACACAAGACATC	
ZO-2	F:GGAAAGATGGAAGGGATGGATGATG	XM_017341705.1
	R:TCACTGATGAGGCGGCTGTC	

### 2.11 Determination of SCFAs in cecum contents

Short-chain fatty acids (SCFAs) were analyzed using a Waters Acquity UPLC-AB SCIEX 5500 QQQ-MS system equipped with two columns: an Acquity UPLC BEH C18 column and an Acquity UPLC HSS T3 column. The analytical procedure was performed as follows: A precisely weighed sample was transferred to a 10 mL centrifuge tube and mixed with 5 mL of extraction solution (methanol:water:formic acid, 15:4:1, v/v/v, containing 0.5% butylated hydroxytoluene). The mixture was vortexed for 1 min, followed by ultrasonication for 30 min. After standing at −40 °C for 60 min, the sample was centrifuged at 12,000 rpm for 10 min. The supernatant was collected for subsequent analysis. Before solid-phase extraction (SPE), the SPE column was activated sequentially with 3 mL of water and 3 mL of methanol. The collected supernatant was then loaded onto the SPE column at a flow rate of ≤1 mL/min. The column was washed with 3 mL of water and 10% methanol, followed by elution with 1 mL of methanol. The eluate was concentrated to dryness using a concentrator and reconstituted in 0.60 mL of 80% methanol. After vortex mixing for 1 min and centrifugation at 12,000 rpm for 10 min, the final supernatant was subjected to UPLC-MS/MS analysis.

### 2.12 16S rRNA sequencing of gut microbiota

In this study, microbial community analysis was performed using 16S rRNA gene sequencing. The V3-V4 hypervariable regions of the 16S rRNA gene were amplified by PCR with universal primers. The resulting sequences were taxonomically classified by comparison to reference databases to determine microbial composition and infer functional profiles. Total genomic DNA was extracted from duodenum and cecum samples using the HiPure Stool DNA Kit (Magen, Guangzhou, China) following the manufacturer’s protocol. DNA quality was assessed through 1.5% ~ 2% agarose gel electrophoresis, while concentration and purity were determined using a NanoDrop One/OneC spectrophotometer (Thermo Fisher Scientific, Waltham, MA, USA). The V3-V4 hypervariable regions of bacterial 16S rRNA genes were amplified using barcoded primers 341F (5′-CCTACGGGNGG CWGCAG-3′) and 806R (5′-GGACTACHVGGGTATCTAAT-3′). PCR products were purified using AMPure XP Beads (Beckman, CA, USA) and quantified using Qubit 3.0 Fluorometer (Thermo Fisher Scientific). Sequencing libraries were prepared using the Illumina DNA Prep Kit (Illumina, San Diego, CA, USA), with quality assessment performed on an ABI StepOnePlus Real-Time PCR System (Applied Biosystems, Foster City, USA). Finally, paired-end sequencing (2 × 250 bp) was conducted on the NovaSeq 6000 platform (Illumina) using the NovaSeq 6000 S2 Reagent Kit v1.5.

### 2.13 Statistical analysis

All experimental data, including immune organ indices, immune factors, antioxidant parameters, and morphological measurements, were expressed as mean ± standard error of the mean (SEM). Normality of data distribution was confirmed using appropriate statistical tests. Statistical analyses were performed using SPSS version 20.0 (IBM Corporation, Chicago, IL, USA). One-way analysis of variance (ANOVA) followed by Tukey’s *post hoc* test was employed to assess intergroup differences. Statistical significance was set at *p* < 0.05. Graphical representations were generated using GraphPad Prism version 9.0 (GraphPad Software, San Diego, CA, USA). Gut microbiome analysis and visualization were performed using R software with specific packages for data processing and graphical representation.

## 3 Results

### 3.1 Growth performance

The effects of PCP on growth performance in Hyla rabbits are summarized in Table 3. Significant improvements in body weight (BW) were observed in both the PL and PM groups compared to the control group at 21 and 42 days of age (*p* < 0.05). Specifically, the PL group showed significant increases in ADG (*p* < 0.01) and ADFI (*p* < 0.05) at 21 days compared to the CON group. Throughout the experimental period (1–42 days), PL, PM, and PH groups demonstrated significantly enhanced ADG (*p* < 0.001) and ADFI (*p* < 0.05) relative to the CON group; meanwhile, the ADG in the PL group was significantly higher than that in the PH group (*p* < 0.01). Notably, the feed conversion ratio (FCR) in the PL group was significantly lower than that of the CON group (*p* < 0.05).

**Table 3:** Effects of PCP on growth performance in weaned rabbits.

Item	Group				SEM	*p-*value
	CON	PL	PM	PH		
Days 1–21						
IBW, kg	0.71	0.71	0.71	0.71	0.10	0.898
FBW, kg	1.34^a^	1.63^b^	1.51^ab^	1.43^ab^	0.14	0.013
ADG, g/d	32.51^a^	41.94^b^	35.94^ab^	34.95^ab^	4.71	0.005
ADFI, g	146.94^a^	143.40^b^	144.20^ab^	145.06^ab^	1.52	0.015
FCR	4.20	3.72	4.08	4.16	0.22	0.247
Days 22–42						
FBW, kg	2.32^a^	2.49^b^	2.49^ab^	2.45^ab^	0.08	0.005
ADG, g/d	32.07	37.59	36.72	33.15	3.45	0.135
ADFI, g	149.08	149.08	146.03	148.85	2.15	0.053
FCR	4.39^a^	3.71^b^	3.83^ab^	4.20^ab^	0.32	0.029
Days 1–42						
ADG, g/d	32.29^a^	39.77^c^	36.33^bc^	33.35^b^	4.06	<0.001
ADFI, g	150.39^a^	144.51^c^	146.20^bc^	147.87^bc^	2.51	0.027
FCR	4.43^a^	3.68^b^	4.02^ab^	4.23^ab^	0.28	0.038

Different superscript letters (a–c) within the same row indicate statistically significant differences (*n* = 6, *p* < 0.05). The following tables are identical. IBW, initial body weight; FBW, final body weight; ADG, average daily gain; ADFI, average daily feed intake; FCR, feed conversion ratio (feed:gain, g:g).

### 3.2 Serum biochemistry

The effects of PCP on serum biochemical parameters in Hyla rabbits are presented in Table 4. Significant alterations were observed in several biochemical indices between the treatment and control groups. Both PL and PM groups exhibited significantly elevated levels of TP, ALB, and GLU compared to the control group (*p* < 0.05), while demonstrating reduced TG levels (*p* < 0.05). Extremely, the PL group showed significant decreases in TC (*p* < 0.05) compared to the control group.

**Table 4:** Effects of PCP on serum biochemistry in weaned rabbits.

Item	Group				SEM	*p-*value
	CON	PL	PM	PH		
TP (g/L)	51.96^a^	59.50^b^	59.17^b^	57.03^ab^	3.48	0.018
ALB (g/L)	31.79^a^	38.51^b^	38.35^b^	36.45^ab^	3.13	0.027
GLB (g/L)	20.17	21.14	20.83	20.57	0.41	0.983
ALP (U/L)	213.68	183.84	195.23	199.01	12.32	0.219
LDH (U/L)	353.46	303.06	304.66	307.34	33.11	0.062
GLU (mmol/L)	5.34^a^	6.31^b^	6.49^b^	5.64^ab^	0.54	0.038
BUN (mmol/L)	6.16	5.54	5.75	5.69	0.27	0.086
TC (mmol/L)	2.06^a^	1.56^b^	1.64^ab^	1.74^ab^	0.22	0.052
TG (mmol/L)	1.92^a^	1.02^b^	1.05^b^	1.27^ab^	0.42	0.031

TP, total protein; ALB, albumin; GLB, globulin; ALP, alkaline phosphatase; LDH, lactate dehydrogenase; GLU, glucose; BUN, blood urea nitrogen; TC, total cholesterol; TG, triglyceride. Different superscript letters (a-c) within the same row indicate statistically significant differences (*n* = 6, *p* < 0.05).

### 3.3 Antioxidant function

The effects of PCP on antioxidant parameters in Hyla rabbits are summarized in Table 5. Compared to the control group, the PL group showed significantly enhanced serum antioxidant capacity, with increased activities of T-AOC (*p* < 0.05), CAT (*p* < 0.05), and GSH-PX (*p* < 0.01). Both PL and PM groups exhibited significantly lower serum MDA levels than the control group (*p* < 0.01), with the PL group showing an additional significant reduction compared to the PH group (*p* < 0.01). In liver tissue, T-AOC and GSH-PX activities were significantly elevated in both PL and PM groups compared to the control group (*p* < 0.01). The PL group demonstrated significantly higher CAT content (*p* < 0.05) and reduced MDA levels (*p* < 0.01), consistent with the PM group’s significant decrease in liver MDA (*p* < 0.01).

**Table 5:** Effects of PCP on antioxidant function in weaned rabbits.

Item	Group				SEM	*p-*value
	CON	PL	PM	PH		
Serum						
MDA (nmol/mL)	13.89^a^	6.67^c^	8.33^bc^	11.94^ab^	3.30	0.004
T-AOC (mmol/mL)	0.23^a^	0.73^b^	0.31^ab^	0.28^ab^	0.23	0.013
CAT (U/mL)	8.49^a^	13.28^b^	9.86^ab^	9.59^ab^	2.07	0.011
SOD (U/mL)	92.23	99.77	95.16	96.3	1.52	0.179
GSH-PX (U/mL)	356.72^a^	388.25^b^	375.83^ab^	370.02^ab^	13.09	0.006
Liver						
MDA (nmol/mgprot)	18.06^a^	11.28^b^	11.37^b^	14.06^ab^	3.18	0.007
T-AOC (mmol/gprot)	5.87^a^	8.27^b^	8.03^b^	6.94^ab^	1.10	0.004
CAT (U/mgprot)	6.74^a^	10.7^b^	8.63^ab^	8.05^ab^	1.65	0.038
SOD (U/mgprot)	104.72	113.61	113.61	108.03	3.77	0.092
GSH-PX (U/mgprot)	113.7^a^	143.47^b^	149.36^b^	133.77^ab^	15.63	0.009

MDA, Malondialdehyde; T-AOC, total antioxidant capacity; CAT, catalase; SOD, Superoxide dismutase; GSH-PX, Glutathione peroxidase. Different superscript letters (a-c) within the same row indicate statistically significant differences (*n* = 6, *p* < 0.05).

### 3.4 Immune function

The immune organ indices are shown in Table 6. The PL group demonstrated a markedly higher thymus index compared to the control group (*p* < 0.01). Conversely, both PL and PM groups exhibited significantly reduced liver indices relative to the control group (*p* < 0.01).

**Table 6:** Effects of PCP on immune organ indices in Hyla rabbits.

Item %	Group				SEM	*p-*value
	CON	PL	PM	PH		
Thymus index	0.15^a^	0.27^b^	0.23^ab^	0.21^ab^	0.05	0.003
Spleen index	0.07^a^	0.12^b^	0.11^b^	0.09^ab^	0.02	0.002
Liver index	2.98^a^	2.27^b^	2.35^b^	2.58^ab^	0.32	0.009
Kidney index	0.51	0.63	0.58	0.56	0.05	0.129
Round vesicle index	0.11	0.15	0.14	0.14	0.02	0.037

Different superscript letters (a-c) within the same row indicate statistically significant differences (*n* = 6, *p* < 0.05).

**Figure 1:**
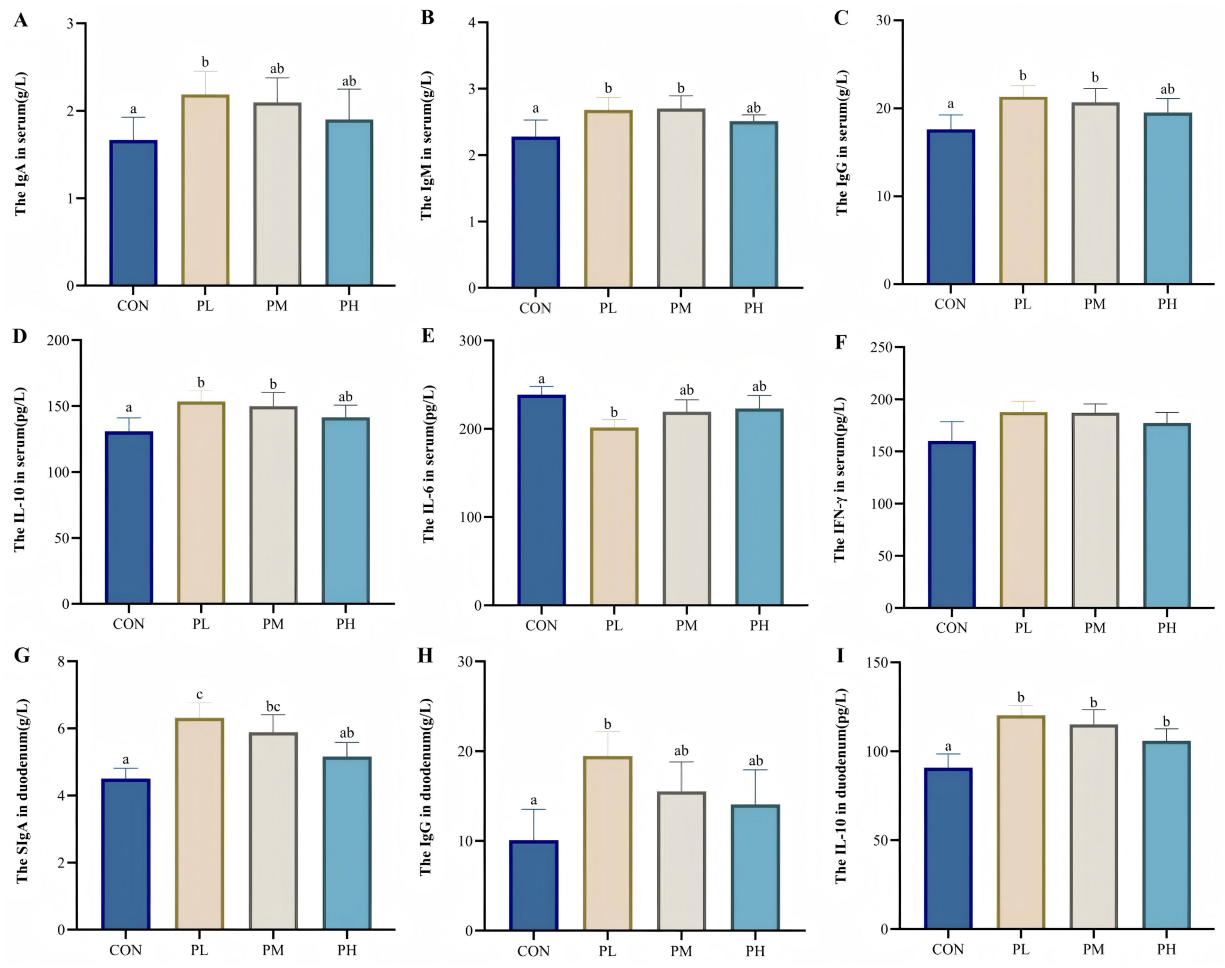
**(A–F)** show the effect of PCP on immune levels in serum, G-I show the effect of PCP on immune factors in the duodenum: **(A)** IgA, **(B)** IgM, **(C)** IgG, **(D)** IL-10, **(E)** IL-6, **(F)** IFN-γ, **(G)** SIgA, **(H)** IgG, **(I)** IgG, IL-10. Data were given as mean ± SEM. (*n* = 6). Bars in the figure without the same superscripts (a–c) differ significantly (*p* < 0.05).

ELISA results of serum and duodenum tissue samples are presented in Figure 1. Both PL and PM groups showed significant increases in serum immunoglobulin and cytokine levels compared to the control group, including IgM (*p* < 0.01, Figure 1B), IgG (*p* < 0.001, Figure 1C), and IL-10 (*p* < 0.01, Figure 1D). The PL group specifically demonstrated higher IgA content (*p* < 0.05, Figure 1A) and reduced IL-6 levels (*p* < 0.05, Figure 1C). In duodenum tissue, SIgA levels were significantly elevated in both PL and PM groups compared to the control group (*p* < 0.01, Figure 1G), with the PL group showing additional increased relative to the PH group (*p* < 0.05, Figure 1G). Furthermore, the PL group exhibited increased IgG activity (*p* < 0.05, Figure 1H), while all PCP-treated groups showed enhanced IL-10 content (*p* < 0.01, Figure 1I) compared to the control group.

### 3.5 Duodenum and cecum morphology

**Figure 2:**
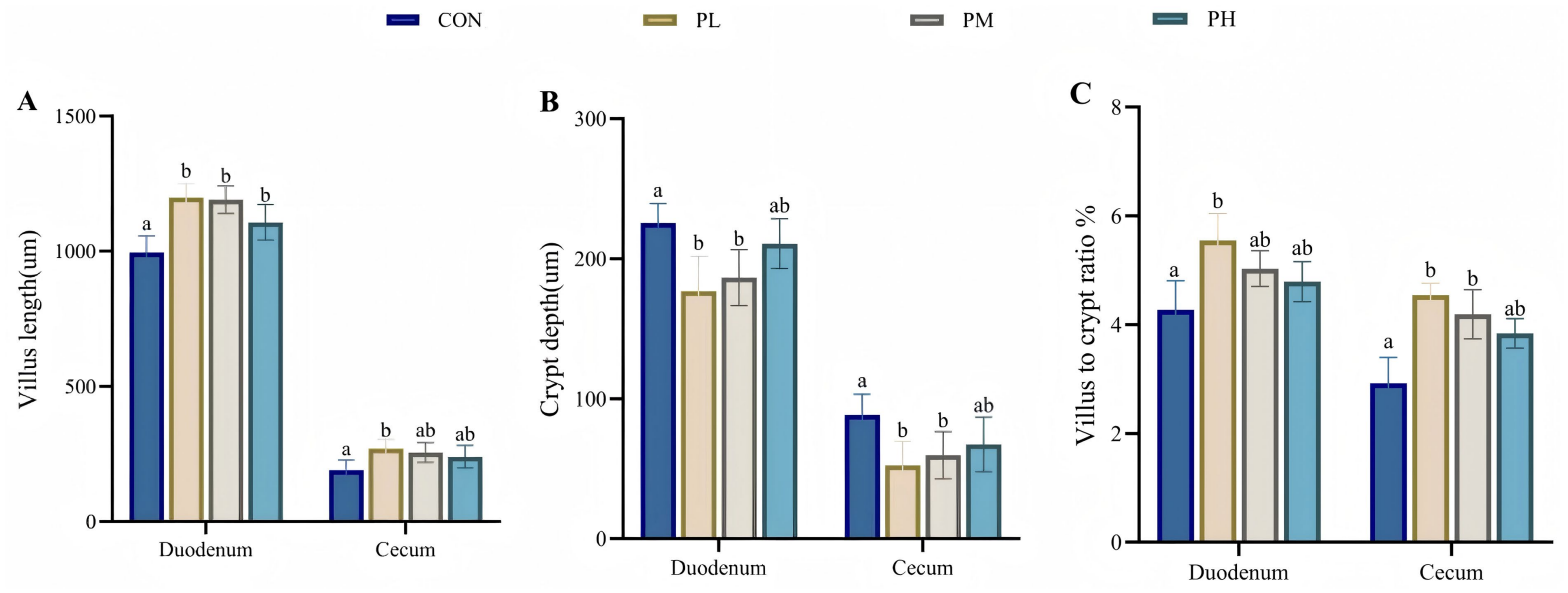
Effects of PCP on duodenum and cecum intestinal morphology: **(A)** Villus length. **(B)** Crypt depth. **(C)** Villus to crypt ratio. Data were given as mean ± SEM (*n* = 6). Bars in the figure without the same superscripts (a–c) differ significantly (*p* < 0.05).

The effects of PCP on intestinal morphology in the duodenum and cecum are illustrated in Figure 2. All PCP-treated groups demonstrated significantly increased villus height in duodenum compared to the control group (*p* < 0.001, Figure 2A). Specifically, the PL group showed enhanced villus length in cecum (*p* < 0.05, Figure 2A) and improved villus-to-crypt ratio in duodenum (*p* < 0.01, Figure 2C). Both PL and PM groups exhibited reduced crypt depth in duodenum and cecum compared to the control group (*p* < 0.01, Figure 2B), with the cecum samples showing significantly increased villus-to-crypt ratio (*p* < 0.01, Figure 2C).

### 3.6 Intestinal permeability

**Figure 3:**
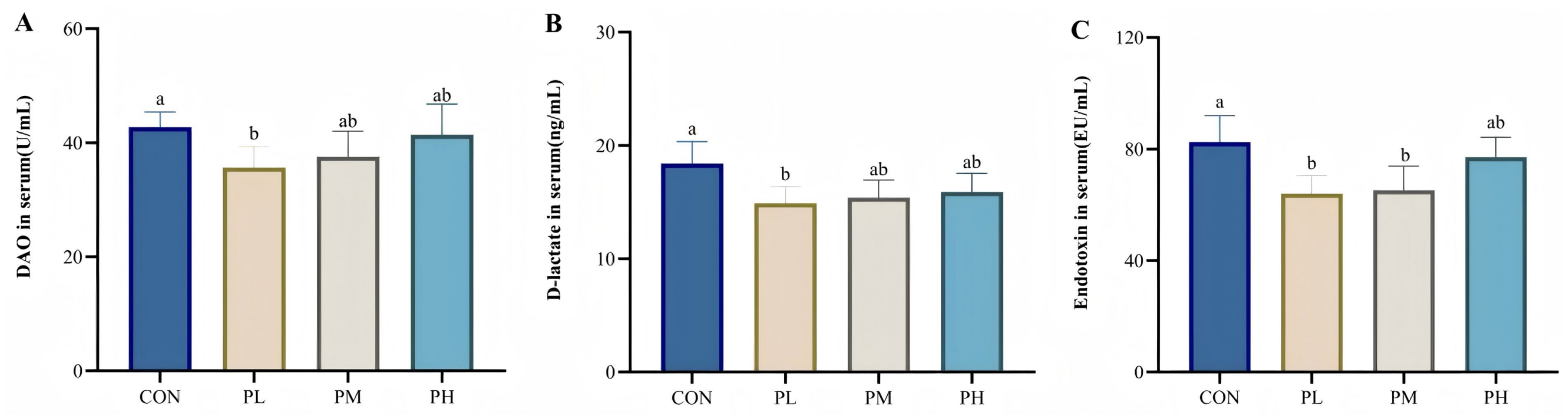
Effects of PCP on intestinal permeability: **(A)** DAO. **(B)** D-lactate. **(C)** Endotoxin. Data were given as mean ± SEM (*n* = 6). Bars in the figure without the same superscripts (a–c) differ significantly (*p* < 0.05).

The effects of PCP on intestinal permeability are presented in Figure 3. Compared to the control group, the PL group exhibited significantly lower serum concentrations of DAO and D-lactate (*p* < 0.05, Figure 3A; *p* < 0.05, Figure 3B). Similarly, serum endotoxin levels were significantly reduced in both PL and PM groups relative to the CON group (*p* < 0.05, Figure 3C).

### 3.7 Intestinal barrier genes expression

**Figure 4:**
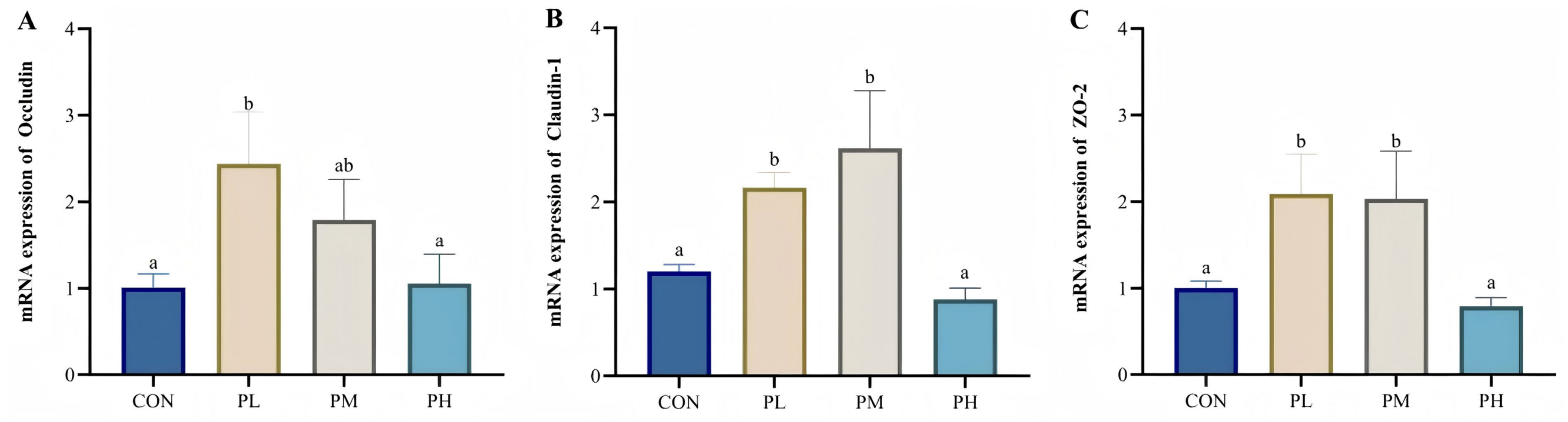
Effects of PCP on the relative mRNA expression of duodenum barrier function genes: **(A)** Occludin. **(B)** Claudin-1. **(C)** ZO-2. Data were given as mean ± SEM (*n* = 6). Bars in the figure without the same superscripts (a–c) differ significantly (*p* < 0.05).

The effects of PCP on intestinal barrier gene expression are illustrated in Figure 4. Compared to the control and PH groups, the PL group showed significantly upregulated Occludin expression (*p* < 0.05, *p* < 0.01; Figure 4A). Similarly, duodenal mucosal expression of Claudin-1 (*p* < 0.05; Figure 4B) and ZO-1 (*p* < 0.05, *p* < 0.01; Figure 4C) was significantly elevated in both PL and PM groups relative to the CON and PH groups.

### 3.8 SCFA analysis of cecum contents

**Figure 5:**
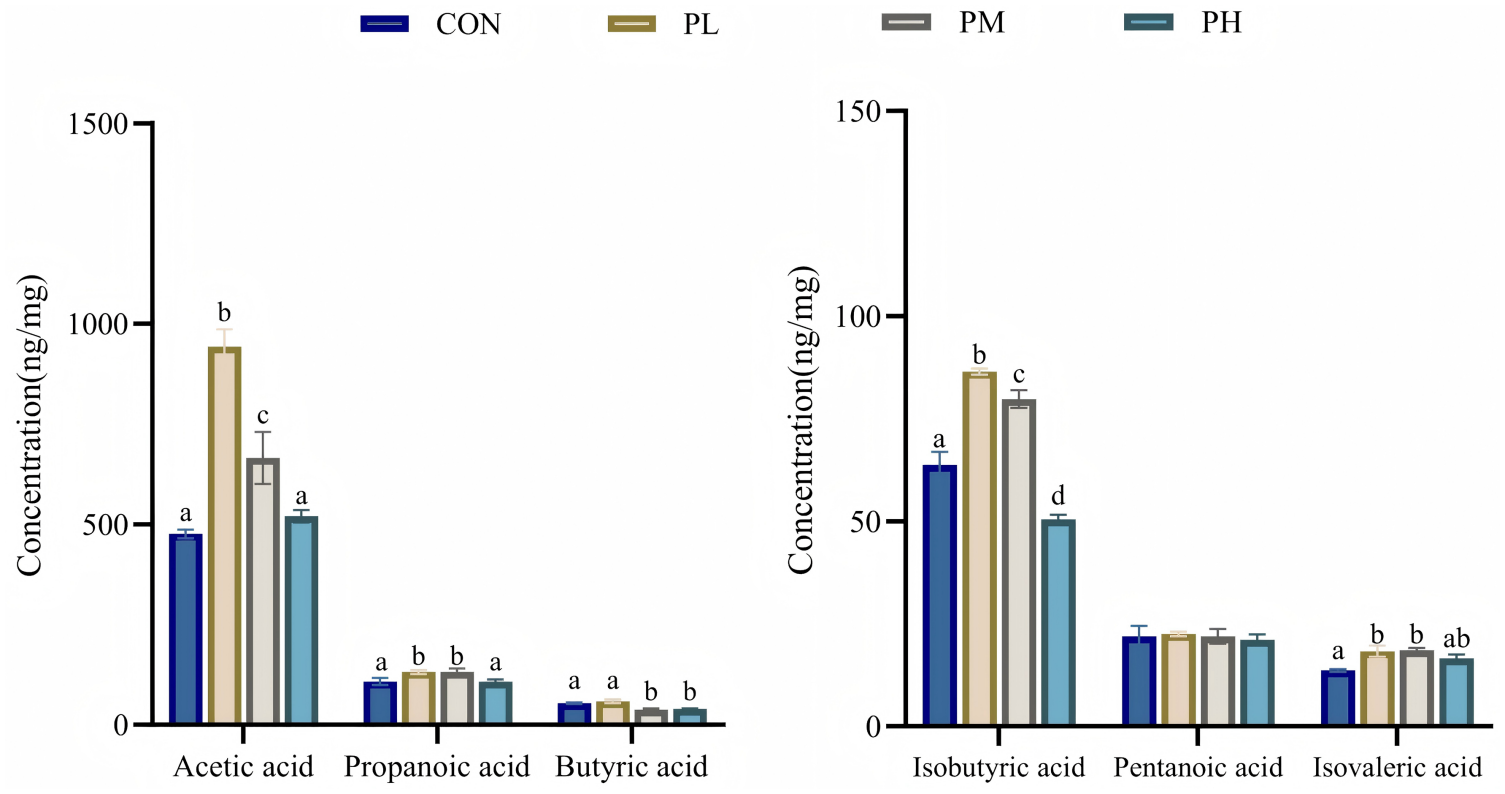
Effects of PCP on SCFA in cecum contents. Data were given as mean ± SEM (*n* = 6). Bars in the figure without the same superscripts (a–c) differ significantly (*p* < 0.05).

The effects of PCP on SCFA content are presented in Figure 5. Compared to the control group, the PL and PM groups exhibited significantly elevated levels of acetic acid (*p* < 0.001), propionic acid (*p* < 0.001), isobutyric acid (*p* < 0.001), and isovaleric acid (*p* < 0.001), with the PL group showing particularly higher acetic and isobutyric acid concentrations relative to other groups. Meanwhile, butyric acid content was comparable between control and PL groups, both significantly exceeding the other two groups (*p* < 0.001).

### 3.9 Gut microbiota composition

#### 3.9.1 Microbiological analysis of duodenum

**Figure 6:**
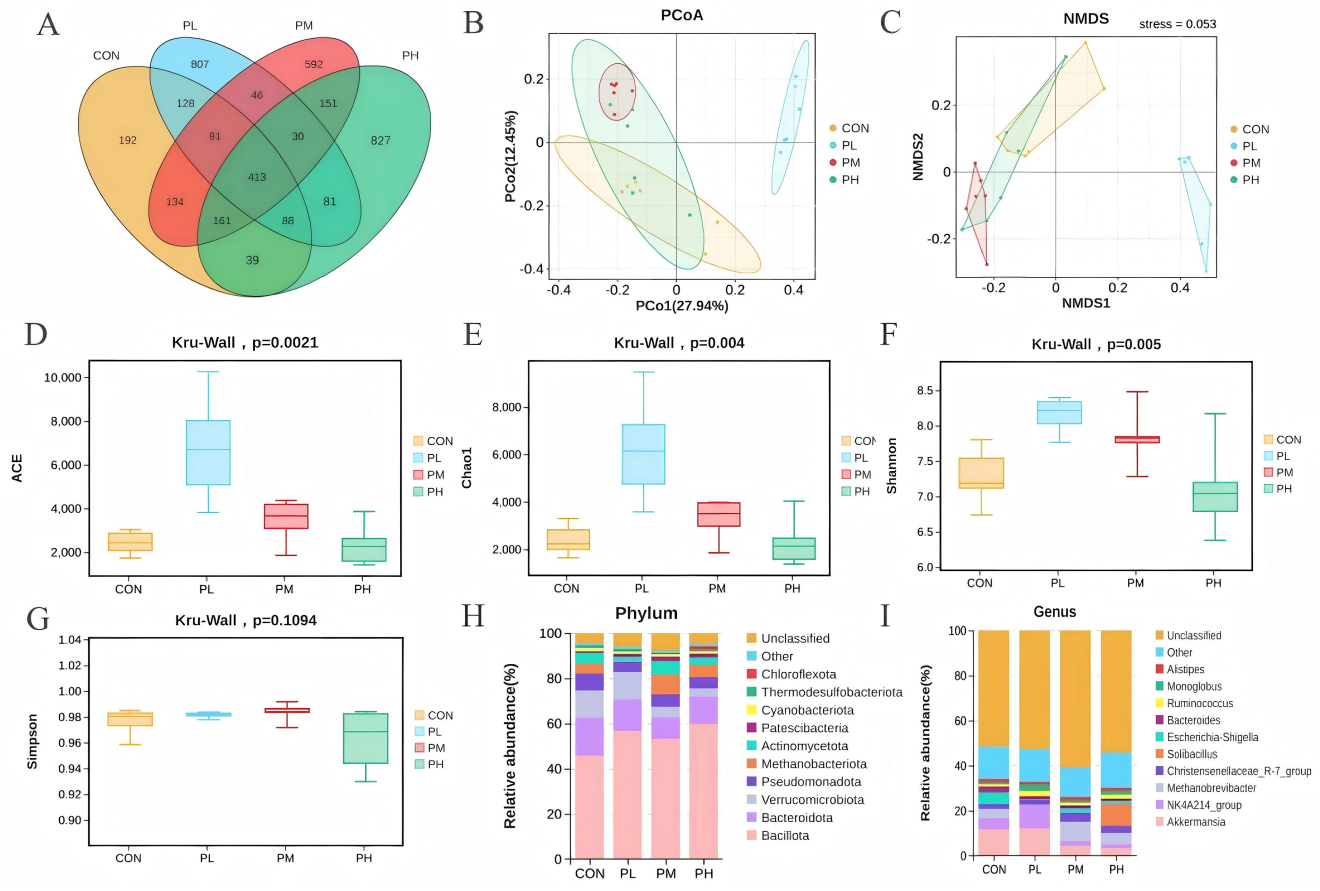
Effect of PCP supplementation on duodenum microbiota diversity. **(A)** Venn diagram; **(B,C)** Intestinal microbial beta diversity analysis (PCoA and NMDS); **(D–G)** Effect of TMAE on intestinal microbial alpha diversity (ACE, Chao1, Shannon and Simpson index); **(H,I)** Colony composition of duodenum microorganisms at phylum and genus levels.

The effects of PCP on duodenal microbiota composition are shown in Figure 6. Venn analysis identified 413 overlapping OTUs among the four groups (Figure 6A). Principal coordinate analysis (PCoA) revealed distinct clustering patterns, with clear separation between the control and PL groups, indicating significant differences in microbial community composition (Figure 6B). Non-metric multidimensional scaling (NMDS) analysis further supported these findings, with a stress value of 0.053 (<0.2), confirming substantial compositional differences between the control and PL groups (Figure 6C). Alpha diversity analysis of poultry intestinal microbiota was conducted using four indices (Figures 6D–G). Species richness, represented by Chao1 and ACE indices, was significantly higher in the PL and PM groups compared to the control group (*p* = 0.0021, Figure 6D; *p* = 0.004, Figure 6E). The Shannon index, indicating community diversity, was significantly lower in the control group (*p* = 0.005, Figure 6F), while no significant differences were observed in Simpson indices (*p* = 0.1094, Figure 6G). To investigate the impact of dietary PCP supplementation on microbial composition, we characterized the microbial communities across dose groups at both phylum and genus taxonomic levels. Microbial community composition analysis at the phylum level revealed *Bacillota*, *Bacteroidota,* and *Verrucomicrobiota* as dominant phyla across all duodenum samples, and the relative abundance of both *Bacillota* and *Bacteroidota* was significantly higher in all PCP groups (Figure 6H). At the genus level, *Akkermansia* and *NK4A214_group* showed increased relative abundance in the PL group compared to the control, PM, and PH groups. However, no significant differences in overall microbial richness were observed at the genus level among groups (Figure 6I).

#### 3.9.2 Microbiological analysis of cecum

**Figure 7:**
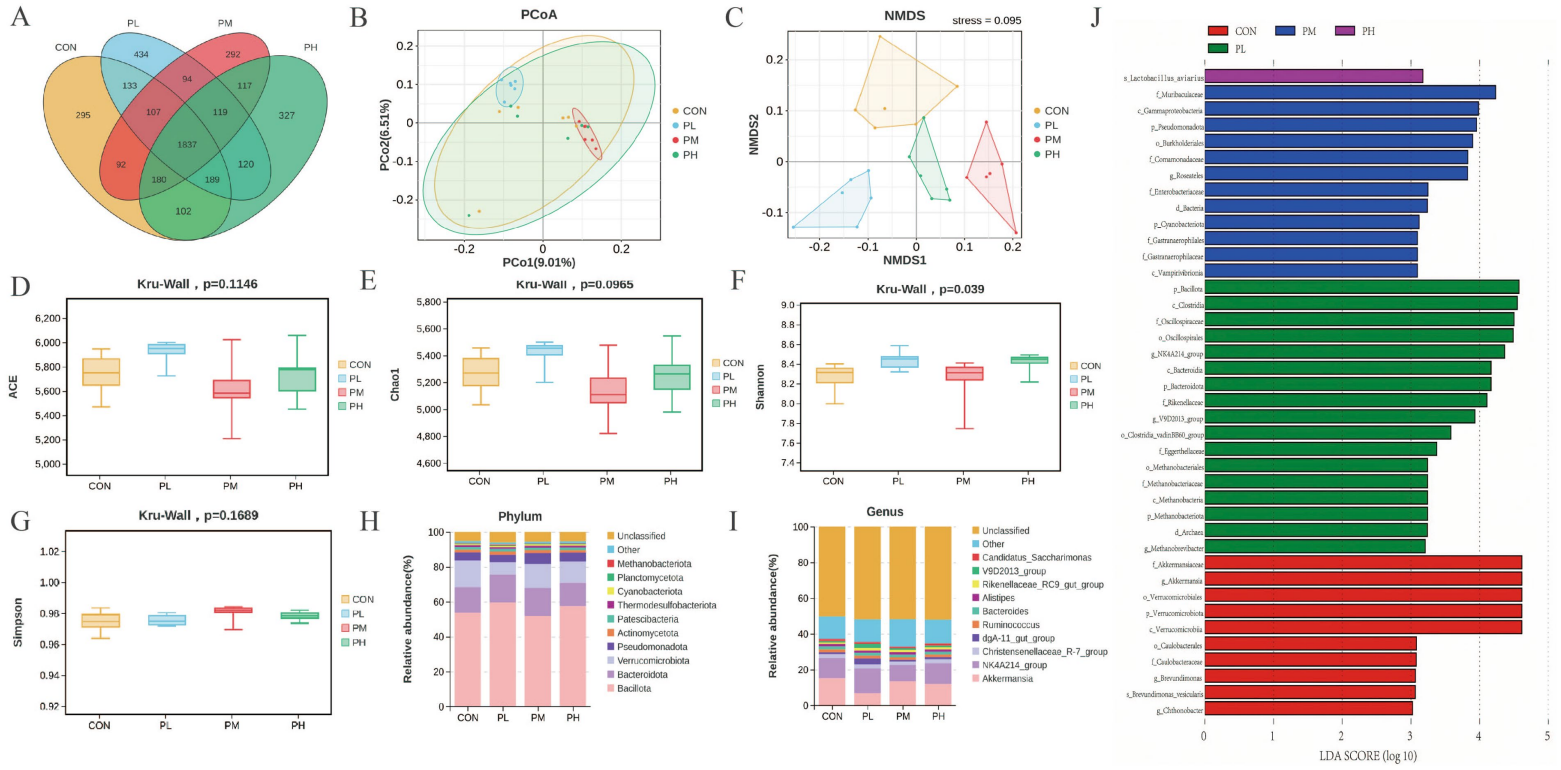
Effect of PCP supplementation on cecum microbiota diversity. **(A)** Venn diagram; **(B,C)** Intestinal microbial beta diversity analysis (PCoA and NMDS); **(D–G)** Effect of TMAE on intestinal microbial alpha diversity (ACE, Chao1, Shannon and Simpson index); **(H,I)** Colony composition of cecum microorganisms at phylum and genus levels; **(J)** LEfSe histogram data showed the LDA scores (>3.0) computed for microbial community features at the OTU levels.

The effects of PCP on cecal microbiota composition are presented in Figure 7. Venn analysis revealed 1837 shared OTUs among the four groups (Figure 7A). PCoA and NMDS analyses revealed distinct clustering patterns, with significant separation between groups, particularly between the PL and PM groups compared to the control group, despite some sample overlap, confirming effective grouping and clear microbial community differentiation (Figures 7B,C). Alpha diversity analysis showed no significant differences in the other three indices, except for an increased Shannon index in PCP-treated groups (*p* = 0.039, Figure 7F). At the phylum level, cecal microbial composition resembled duodenal patterns across all groups (Figure 7H). However, genus-level analysis revealed decreased *Akkermansia* abundance in all PCP groups compared to the CON group, while *NK4A214_group* showed significant enrichment in the PL group (Figure 7I). Furthermore, LEfSe analysis (LDA score > 3.0) was performed to identify microbial taxa contributing significantly to community differences. *Bacillota*, *Clostridia*, and *OscillospHylaceae* dominated the PL group; *Akkermansiaceae* and *Verrucomicrobiota* characterized the PM group; and *Muribaculaceae*, *Gammaproteobacteria*, *Pseudomonadota*, and Burkholderiales were predominant in the control group (Figure 7J).

## 4 Discussion

Previous studies have demonstrated the beneficial effects of PCP in livestock and poultry production. Our findings further indicate that dietary supplementation with optimal PCP concentrations enhances growth performance in Hyla rabbits, as evidenced by increased ADG and ADFI, along with improved feed conversion ratio. Specifically, the 0.1% PCP supplementation showed optimal growth-promoting effects, consistent with existing literature on the growth-enhancing properties of polysaccharides in weaned rabbits (15).

Serum biomarkers serve as crucial indicators of animal growth, immunity, and metabolic status (16). The content of TP, ALB, GLB, and BUN in serum reflects protein metabolism and immune function (17). Serum TC and TG, as primary lipid components, indicate lipid utilization and overall health status, with reduced levels potentially signifying enhanced lipolysis (18). GLU, the primary energy source, not only reflects health status but also improves growth performance when moderately elevated (19). Previous studies have demonstrated the beneficial effects of *Poria cocos* extracts on serum parameters. Yang et al. (20) reported that dietary supplementation with 0.1–0.2% *Poria cocos* extract significantly increased serum TP and ALB levels while reducing TG in meat rabbits. Similarly, Wu (21) observed elevated GLU levels following supplementation with 80–160 mg/kg PCP. Consistent with these findings, our study revealed that dietary supplementation with 0.1–0.2% PCP significantly increased TP, ALB, and GLU levels in Hyla rabbits. Notably, the 0.1% PCP supplementation significantly reduced TG levels, which is in line with the above-mentioned findings.

Antioxidant capacity serves as a critical indicator of an organism’s health status, reflecting the balance between oxidative and antioxidant systems in regulating free radical metabolism (22). Herbal polysaccharides exert antioxidant effects primarily through free radical scavenging and modulation of antioxidant enzyme activities (23). Key components of the antioxidant defense system, including GSH-Px, SOD, and CAT, maintain redox homeostasis and protect against oxidative damage (24). T-AOC represents a comprehensive biochemical assessment of an organism’s overall antioxidant potential, reflecting the organism’s overall ability to neutralize reactive oxygen and nitrogen species (ROS/RNS) (25). MDA, as the terminal product of free radical-mediated lipid peroxidation, serves as a critical biomarker for assessing oxidative damage to cellular components (26). PCP demonstrates significant antioxidant properties, protecting against free radical-induced cellular damage and enhancing endogenous antioxidant systems (27, 28). Our findings revealed that PCP supplementation increased T-AOC, CAT, and GSH-Px levels in both serum and liver tissues of Hyla rabbits. These results align with Sun’s (29) observations in piglets. Furthermore, 0.1–0.2% PCP supplementation significantly reduced MDA levels in serum and liver, consistent with Wu’s (21) findings, demonstrating PCP’s capacity to enhance antioxidant status.

Immune organ indices and serum immunity parameters serve as reliable indicators of health status in rabbits (30). The thymus, as the central organ for cellular immunity, and the spleen, the largest peripheral immune organ with unique immunological functions, along with the rabbit-specific lymphoid structure (round vesicle), collectively reflect the immune status (31, 32). In this study, dietary supplementation with 0.1% PCP significantly increased thymus and spleen indices, while 0.2% PCP specifically enhanced spleen index, consistent with Wang et al.’s (33) findings. Immunoglobulins play crucial roles in immune defense: IgA mediates mucosal immunity, IgG provides systemic protection against infections, and IgM serves as the primary antibody in early immune responses (34, 35). Cytokines serve as essential cellular messengers, playing critical roles in immune responses (36). Specifically, the pro-inflammatory cytokine IL-6 activates immune cells and promotes inflammatory responses, whereas the anti-inflammatory cytokine IL-10 mitigates inflammation and modulates immune cell activity (37). Previous studies have demonstrated *Poria cocos* polysaccharides’ capacity to enhance both specific and non-specific immunity (38). This may be due to its potential signalling pathways, which exert anti-inflammatory and immunomodulatory effects. Research by Fang et al. (39) suggested that PCP may enhance immune function in immunosuppressed mice through inhibition of the BTLA/HVEM pathway. Similarly, Sun et al. (40) reported that PCP induces innate immune regulation via activation of the TLR2/4/MyD88/NF-κB signaling pathway in M1-polarized RAW264.7 cells. In addition, Cai et al. (41) demonstrated that PCP suppresses the production of inflammatory mediators by downregulating protein expression in the TLR4/NF-κB pathway, thereby reducing the generation and infiltration of inflammatory cells and subsequently alleviating inflammatory damage in lung tissue. Previous studies have demonstrated the immunomodulatory effects of PCP in animals. Zhang et al. (12) reported that PCP supplementation significantly increased serum IgA, IgG, and IL-10 levels, moderately elevated IgM, and reduced TNF-*α* and IL-6 levels. Sun (29) observed that 0.05–0.2% PCP administration enhanced serum IgG, IgM, IgA, IL-2, and IFN-γ levels of Piglets. Consistent with these findings, our results showed that dietary PCP elevated serum IgA, IgG, IgM, and IL-10 while reducing IL-6 levels in Hyla rabbits. However, unlike previous reports, we observed no significant effect on IFN-γ levels, suggesting potential species-specific differences in PCP’s immunomodulatory activity.

The intestinal tract serves as the primary site for digestion and nutrient absorption, with its morphological features directly reflecting intestinal health and nutrient utilization efficiency (42). Villus height, crypt depth, and their ratio (V/C) are critical indicators of intestinal structural and functional integrity. Increased villus height and V/C ratio correlate with enhanced absorptive capacity, while deeper crypts may indicate accelerated epithelial cell turnover, potentially reflecting inflammatory responses or tissue repair (43, 44). Intestinal barrier integrity is essential for maintaining physiological and immune functions (45). Tight junction proteins form the physical barrier, regulating intestinal permeability and preventing bacterial translocation and macromolecule infiltration (46, 47). DAO is a cytoplasmic enzyme in intestinal mucosal cells that enters circulation upon intestinal damage, serving as a damage marker (48). Under normal conditions, the intestinal barrier prevents luminal bacteria and endotoxins from entering the bloodstream. However, barrier dysfunction increases mucosal permeability, allowing endotoxin and D-lactic acid (a bacterial fermentation product) to translocate. Additionally, secretory IgA (sIgA) provides chemical barrier protection against pathogenic invasion (49, 50). Previous studies have demonstrated the protective effects of PCP on intestinal barrier function. Ao et al. (51) reported that PCP administration in diabetic mice reduced serum markers of intestinal barrier dysfunction and pro-inflammatory cytokines while upregulating ZO-1 and occludin expression. Similarly, PCP was shown to mitigate. Cyclophosphamide-induced intestinal mucosal injury in mice through improved colonic physiology, reduced intestinal permeability, inhibited epithelial apoptosis, and enhanced mucosal immunity (52). Duan et al. (14) reported that PCP enhanced intestinal barrier integrity by increasing Occludin and ZO-1 expression while reducing serum levels of endotoxin, DAO, and D-lactate in mice. Chen et al. (53) further demonstrated that dietary supplementation with 600 mg/kg Atractylodes macrocephala PCP complex increased jejunal expression of ZO-1, Claudin-1, and Occludin mRNA in piglets, reduced plasma D-lactate and DAO levels, and enhanced villus height in duodenum, jejunum, and ileum. Consistent with these findings, our study revealed that dietary PCP supplementation enhanced duodenal SIgA, IgG, and IL-10 levels while increasing Occludin, Claudin-1, and ZO-2 gene expression. Additionally, PCP reduced serum DAO, D-lactate, and endotoxin levels, improved villus length and villus-to-crypt ratio in duodenum and cecum, and decreased crypt depth. These results collectively demonstrate that PCP supplementation maintains intestinal health by enhancing tight junction protein expression, improving intestinal immunity, and preserving mechanical barrier function through morphological optimization of intestinal villi and crypts in Hyla rabbits.

The gut microbiota plays crucial physiological roles in nutrient metabolism, energy homeostasis, mucosal barrier maintenance, and immunomodulation (54). Thus, a stable and balanced intestinal microbial ecosystem is essential for maintaining gut health. Increased microbial diversity enhances colonization resistance, limiting pathogenic invasion and improving host defense mechanisms (55). Xu et al. (59) demonstrated that probiotic compound preparation (PCP) restored *Bacillota* abundance to normal levels while enhancing microbial diversity in ADD mice. Consistent with these findings, our study revealed that PCP supplementation significantly increased the diversity and abundance of duodenal and cecal microbiota in the PL group, as evidenced by both alpha and beta diversity analyses. These results suggest that PCP may improve growth performance by modulating intestinal microbiota to enhance disease resistance in rabbits. Studies have established *Bacteroidota* and *Bacillota* (formerly *Firmicutes*) as the dominant phyla in rabbit intestinal *microbiota*, with *Clostridia* (a phylum within *Firmicutes*) serving as primary butyrate producers that benefit animal health (56). PCP treatment significantly increased the Bacteroidetes/Firmicutes ratio and enhanced the relative abundance of immunomodulatory gut bacteria, thereby restoring intestinal microbiota homeostasis in splenic rats (57). Duan et al. (14) demonstrated that PCP administration resulted in *Bacteroidota* and *Bacillota* comprising over 90% of mouse intestinal microbiota, with increased *Muribaculaceae* and *Bacteroides* abundance, suggesting PCP-mediated modulation of intestinal barrier function through microbial composition changes. Our findings align with these observations, showing *Bacillota*, *Bacteroidota*, and *Verrucomicrobiota* as dominant phyla in the rabbit duodenum and cecum, with PCP increasing *Bacillot*a and *Bacteroidota* relative abundance. LEfSe analysis revealed *Bacillota*, *Clostridia*, and *OscillospHylaceae* as signature taxa in the PL group cecum. Petra et al. (58). *Clostridium butyricum* is considered to be a probiotic that degrades *oligosaccharides* in food and regulates the balance of the intestinal microbiota. Xu et al. (59) found that *OscillospHylaceae* are candidates for next-generation *probiotics*. Also produces butyric acid, which significantly improves intestinal epithelial function by promoting the expression of intestinal tight junction proteins. Therefore, 0.1%PCP had a regulatory effect on the microbiota and selectively promoted the growth and reproduction of beneficial gut bacteria, and thus enhanced gut health in rabbits. These results demonstrate that 0.1% PCP supplementation selectively promotes beneficial bacterial growth, modulates microbial composition, and enhances intestinal health in Hyla rabbits.

SCFAs, including acetic acid, pentanoic acid, and butyric acid as major components, are microbial metabolites derived from carbohydrate fermentation in the cecum (60). These compounds serve as crucial mediators between gut microbiota and intestinal mucosa, enhancing nutrient absorption, maintaining epithelial integrity, and providing energy substrates (61). Furthermore, SCFAs exhibit immunomodulatory, antimicrobial, and antitumor properties through regulating immune responses and inducing apoptosis (62, 63). Duan et al. (14) reported significant increases in intestinal acetic acid, propionic acid, isobutyric acid, and isovaleric acid following PCP administration in mice. Lai et al. (64) demonstrated that water-insoluble polysaccharides from *Poria cocos* elevated acetic and butyric acid levels in antibiotic-associated diarrhea in mice. Our findings align with these observations, showing that dietary PCP differentially increased cecal concentrations of acetic acid, propionic acid, butyric acid, isobutyric acid, and isovaleric acid in Hyla rabbits. And *Bacteroidota* predominance in both duodenal and cecal microbiota, coupled with PCP-induced *Bacteroidota* enrichment, likely contributed to enhanced SCFA production. These results suggest that PCP may improve intestinal health by modulating microbial communities to promote SCFA synthesis.

## 5 Conclusion

In conclusion, dietary supplementation with PCP, particularly at 0.1 and 0.2% concentrations, enhances growth performance, serum biochemical parameters, antioxidant status, immune responses, and intestinal barrier integrity in Hyla rabbits. Furthermore, it elevates short-chain fatty acid (SCFA) production. These results indicate that PCP is a beneficial feed additive that supports growth, health, and economic viability in rabbit production.

## Data Availability

The original contributions presented in the study are publicly available. This data can be found here: https://doi.org/10.6084/m9.figshare.30514874.v1. RNA sequencing data have been deposited in NCBI, https://www.ncbi.nlm.nih.gov/sra/PRJNA1357065. BioProject accession number for the referenced sequencing data: PRJNA1357065.
